# An Inversion-Free Method for Finding Positive Definite Solution of a Rational Matrix Equation

**DOI:** 10.1155/2014/560931

**Published:** 2014-08-19

**Authors:** Fazlollah Soleymani, Mahdi Sharifi, Solat Karimi Vanani, Farhad Khaksar Haghani, Adem Kılıçman

**Affiliations:** ^1^Department of Mathematics, Islamic Azad University, Shahrekord Branch, Shahrekord, Iran; ^2^Department of Mathematics and Institute for Mathematical Research, Universiti Putra Malaysia, 43400 Serdang, Malaysia

## Abstract

A new iterative scheme has been constructed for finding minimal solution of a rational matrix equation of the form *X* + A*X ^−1^ *A* = *I*. The new method is inversion-free per computing step. The convergence of the method has been studied and tested via numerical experiments.

## 1. Introduction

In this paper, we will discuss the following nonlinear matrix equation:

(1) $$\begin{array}{c} X+A^{*}X^{-1}A=I, \end{array}$$

where *A* is an *n* × *n* nonsingular complex matrix, *I* is the unit matrix of the appropriate size, and *X* ∈ *C* ^*n*×*n*^ is an unknown Hermitian positive definite (HPD) matrix that should be found. It was proved in [1] that if (1) has an HPD solution, then all its Hermitian solutions are positive definite and, moreover, it has the maximal solution *X*_*L*_ and the minimal solution *X*_*S*_ in the sense that *X*_*S*_ ≤ *X* ≤ *X*_*L*_ for any HPD solution *X*.

A lot of papers have been published regarding the iterative HPD solutions of such nonlinear rational matrix equations in the literature due to their importance in some practical problems arising in control theory, dynamical problems, and so forth (see [2, 3]).

The most common iterative method for finding the maximal solution of (1) is the following fixed-point iteration [4]:

(2) X0=I,Xk+1=I−A∗Xk−1A.

The maximal solution of (1) can be obtained through *X*_*L*_ = *I* − *Y*_*S*_, where *Y*_*S*_ is the minimal solution of the dual equation *Y* + *AY* ^−1^ A* = *I*.

In 2010, Monsalve and Raydan in [5] proposed the following iteration method (also known as Newton's method) for finding the minimal solution:

(3) $$\begin{array}{c} X_{0}=AA^{*},\\ X_{k+1}=X_{k}\left(2I-A^{-*}\left(I-X_{k}\right)A^{-1}X_{k}\right), \end{array}$$

which is an inversion-free scheme. Since, *A* ^−1^ should be computed only once in contrast to the matrix iteration (2). Note that *A* ^−∗^ = *A* ^−1*^, and similar notations are used throughout.

**Remark 1 .**

We remark that there are several other well-known iterative methods for solving (1) rather than Newton's method (3). To the best of our knowledge, the procedure of extending higher-order iterative methods for finding the solution of (1) has not been exploited up to now. Hence, we hope that this interlink among the fields of root-finding and solving (1) may lead to discovering novel and innovative techniques.

The rest of this paper is organized as follows. In Section 2, we develop and analyze a new inversion-free method for finding roots of a special map *F*. In Section 3, we provide some numerical comparisons by employing some experiments in machine precision. Some concluding remarks will be drawn in Section 4.

## 2. A New Iterative Method

An equivalent formulation of (1) is to find an HPD matrix *X* such that *F*(*X*) = 0. Toward this goal, we write *F*(*X*)≔*X* + A*X ^−1^ *A* − *I* = 0. Furthermore, we have

(4) F(X)=A∗X−1A+X−I=A∗X−1A−(I−X)=A∗X−1A−(A−∗−XA−∗)A∗=A∗X−1A−A(A−1A−∗−A−1XA−∗)A∗=(A−1XA−∗)−1−A(A−1A−∗−A−1XA−∗)A∗.

Now, using a change of variable as *Z* = *A* ^−1^ *XA* ^−∗^, we could simplify (4) as follows:

(5) $$\begin{array}{c} G\left(Z\right)=Z^{-1}-A\left(A^{-1}A^{-*}-Z\right)A^{*}=0. \end{array}$$

In order to obtain an iterative method for finding the minimal HPD solution of (1), it is now enough to solve the well-known matrix equation *Z* ^−1^ − *B* = 0, at which *B* = *A*(*A* ^−1^ *A* ^−∗^ − *Z*)A*. One of such ways to challenge this matrix inversion problem is via applying the Schulz-type iteration methods (see, e.g., [6–10]). Applying Chebyshev's method [11] yields

(6) X0=AA∗,Xk+1=Xk[3I−A−∗(I−Xk)      ×A−1Xk(3I−A−∗(I−Xk)A−1Xk)].

We remark that there is a tight relationship between iterative methods for nonlinear systems and the construction of higher-order methods for matrix equations ([12, 13]).

The matrix iteration (6) requires *A* ^−1^ to be computed only once at the beginning of the iteration and this makes the iterative method fall in the category of inversion-free algorithms for solving (1).

In the meantime, it is easy to show that the zeros of the map *G*(*Z*) = *Z* ^−1^ − *B* are equal to the zeros of the map *F*(*X*) = *X* ^−1^ − *H*, wherein *H* = *A* ^−∗^(*I* − *X*)*A* ^−1^. To be more precise, we are finding the inverse of the matrix *H* which matches the minimal HPD solution of (1).

**Remark 2 .**

Following Remark 1, we applied Chebyshev's method for (1) in this work and will study its theoretical behavior. The extension of the other well-known root-finding schemes for finding the minimal solution of (1) will remain for future studies.

**Lemma 3 .**

The proposed method (6) produces a sequence of Hermitian matrices using the Hermitian initial matrix *X*_0_ = AA*.

**Proof**

The initial matrix AA* is Hermitian, and *H*_*k*_ = *A* ^−∗^(*I* − *X*_*k*_)*A* ^−1^. Thus, *H*_0_ = *A* ^−∗^ *A* ^−1^ − *A* ^−∗^ AA*A ^−1^ is also Hermitian; that is, *H*_0_* = *H*_0_. Now using inductive argument, we have

(7) (X1)∗=(X0[3I−H0X0(3I−H0X0)])∗=(3X0−3X0H0X0+[X0H0X0H0X0])∗=3X0−3X0H0X0+X0H0X0H0X0=X1.

By considering (*X*_*l*_)* = *X*_*l*_, (*l* ≥ *k*) we now show that

(8) (Xl+1)∗=(Xl[3I−HlXl(3I−HlXl)])∗=(3Xl−3XlHlXl+[XlHlXlHlXl])∗=3Xl−3XlHlXl+XlHlXlHlXl=Xl+1.

Note that *H*_*l*_ = (*H*_*l*_)* has been used in (8). Now the conclusion holds for any *l* + 1. Thus, the proof is complete.

**Theorem 4 .**

By considering that *A* and *X*_*k*_ are nonsingular matrices, the sequence {*X*_*k*_} generated by (6) is convergent to the minimal solution using the initial matrix *X*_0_ = AA*.

**Proof**

Let us consider *H*_*k*_ = *A* ^−∗^(*I* − *X*_*k*_)*A* ^−1^. We therefore have

(9) I−HkXk+1=I−[A−∗(I−Xk)A−1]  ×(Xk[3I−A−∗(I−Xk)A−1Xk      ×(3I−A−∗(I−Xk)A−1Xk)])=(I−HkXk)3.

Taking a generic matrix operator norm from both sides of (9), we obtain

(10) $$\begin{array}{c} ||I-H_{k}X_{k+1}||\leq {||I-H_{k}X_{k}||}^{3}. \end{array}$$

On the other hand, Chebyshev's method for matrix inversion problem is convergent if the initial approximation reads ||*I* − *HX*_0_|| < 1. That is to say, ||*I* − [*A* ^−∗^(*I* − *X*)*A* ^−1^]*X*_0_|| < 1. This together with the initial matrix *X*_0_ = AA* gives

(11) $$\begin{array}{c} ||A^{-*}XA^{*}||<1, \end{array}$$

which is true when *X* is the minimal HPD solution of (1).

Note that since

(12) 0<XS=I−A∗XS−1A=A∗[A−∗A−1−XS−1]A=A∗[X0−1−XS−1]A,

we obtain that *X*_0_^−1^ > *X*_*S*_^−1^; thus *X*_0_ < *X*_*S*_. And subsequently using* mathematical induction*, it would be observed that {*X*_*k*_} tends to *X*_*S*_.

The only problem that happens in this process is the fact that the convergence order is *q*-linear. In fact, although Chebyshev's method for matrix inversion has third local order of convergence, this rate will not be preserved for finding the minimal HPD solution of (1).

The reason is that the matrix *H*, which we must compute its inverse by Chebyshev's method, is dependent on the *X* itself. That is to say, the unknown is located in the essence of the matrix *H* = *A* ^−∗^(*I* − *X*)*A* ^−1^.

**Theorem 5 .**

The sequence of matrices produced by (6) satisfies the following error inequality:

(13) $$\begin{array}{c} ||X_{k+1}-X_{S}||\leq ||\lambda_{k}||||X_{k}-X_{S}||, \end{array}$$

where *λ*_*k*_ = −*X*_*S*_^−1^ *δ*_*k*_ + *X*_*k*_ *X*_*S*_^2^ *δ*_*k*_ + 3*Y*_*k*_ − *X*_*k*_ *X*_*S*_^−1^ *Y*_*k*_ − *Y*_*k*_ *X*_*S*_^−1^ *X*_*k*_ + *Y*_*k*_ *A* ^−∗^ *A* ^−1^ *X*_*k*_ and *Y*_*k*_ = *X*_*k*_ *A* ^−∗^ *A* ^−1^ *X*_*k*_.

**Proof**

First since lim⁡_*k*→*∞*_ *X*_*k*_ = *X*_*S*_ and by using (6) we have

(14) Xk+1−XS =Xk[3I−A−∗(I−Xk)A−1Xk    ×(3I−A−∗(I−Xk)A−1Xk)]−XS =[3Xk−3XkA−∗(I−Xk)A−1Xk+XkA−∗   ×(I−Xk)A−1XkA−∗(I−Xk)A−1Xk]−XS =[3Xk−3XkA−∗((I−XS)−(Xk−XS))A−1Xk   +XkA−∗((I−XS)−(Xk−XS))A−1XkA−∗   ×((I−XS)−(Xk−XS))A−1Xk]−XS =[3Xk−3XkXS−1Xk+3XkA−∗(Xk−XS)A−1Xk    +Xk(XS−1Xk)2−XkXS−1XkA−∗(Xk−XS)    ×A−1Xk−XkA−∗(Xk−XS)A−1XkXS−1Xk+XkA−∗    ×(Xk−XS)A−1XkA−∗(Xk−XS)A−1Xk]−XS =−[XS−3Xk+3XkXS−1Xk−Xk(XS−1Xk)2]   +3XkA−∗(Xk−XS)A−1Xk+Xk(XS−1Xk)2   −XkXS−1XkA−∗(Xk−XS)A−1Xk   −XkA−∗(Xk−XS)A−1XkXS−1Xk+XkA−∗   ×(Xk−XS)A−1XkA−∗(Xk−XS)A−1Xk.

Note that we have used the fact that (*I* − *X*_*S*_) = A*X_*S*_^−1^ *A*. Relation (14) yields

(15) δk+1=−δkXS−1δk+XkXS2δk2+3XkA−∗(δk)A−1Xk −XkXS−1XkA−∗δkA−1Xk−XkA−∗δkA−1XkXS−1Xk +XkA−∗δkA−1XkA−∗δkA−1Xk=λkδk,

wherein *δ*_*k*_ = *X*_*k*_ − *X*_*S*_ and *λ*_*k*_ = −*X*_*S*_^−1^ *δ*_*k*_ + *X*_*k*_ *X*_*S*_^2^ *δ*_*k*_ + 3*X*_*k*_ *A* ^−∗^ *A* ^−1^ *X*_*k*_ − *X*_*k*_ *X*_*S*_^−1^ *X*_*k*_ *A* ^−∗^ *A* ^−1^ *X*_*k*_ − *X*_*k*_ *A* ^−∗^ *A* ^−1^ *X*_*k*_ *X*_*S*_^−1^ *X*_*k*_ + *X*_*k*_ *A* ^−∗^ *A* ^−1^ *X*_*k*_ *A* ^−∗^ *A* ^−1^ *X*_*k*_. We remark that *δ*_*k*_ *A* ^−1^ *X*_*k*_ = *A* ^−1^ *X*_*k*_ *δ*_*k*_.

Consequently, one has the error inequality (13). This shows the *q*-linear order of convergence for finding the minimal HPD solution of (1). We thus have

(16) $$\begin{array}{c} 0<\underset{k\to \infty }{\mathrm{limsup}}\frac{X_{k+1}-X_{S}}{X_{k}-X_{S}}<1, \end{array}$$

which is guaranteed since

(17) $$\begin{array}{c} 0<\underset{k\to \infty }{\mathrm{limsup}}||\lambda_{k}||<1. \end{array}$$

## 3. Numerical Comparisons

In this section, we mainly investigate the performance of the new method (6) for matrix equation (1). All experiments were run on a Pentium IV computer, using Mathematica 8 [14]. We report the number of required iterations (Iter) for converging. In our implementations, we stop all considered methods when the infinity norm of two successive iterates is less than given tolerance.

Note that recently Zhang in [15] studied a way to accelerate the beginning of such iterative methods for finding the minimal solution of (1) via applying multiple Newton's method for matrix inversion. This technique could be given by

(18) $$\begin{array}{c} X_{k+1}=X_{k}\left(\left(t+1\right)I-tH_{k}X_{k}\right), \end{array}$$

for any 1 ≤ *t* ≤ 2. Subsequently, we could improve the behavior of the new method (6) using (18) as provided in Algorithm 1.

We compare Algorithm 1, denoted by PM, with (2) denoted by M1, (3) denoted by M2, and the method proposed by El-Sayed and Al-Dbiban [16] denoted by M3, which is a modification of the method presented by Zhan in [17], as follows:

(19) $$\begin{array}{c} X_{0}=Y_{0}=I,\\ Y_{k+1}=\left(I-X_{k}\right)Y_{k}+I,\\ X_{k+1}=I-A^{*}Y_{k+1}A. \end{array}$$

**Example 1 (see [18]).**

In this experiment, we compare the results of different methods for finding the minimal solution of (1) when the matrix *A* is defined by

(20) A=(0.370.130.12−0.300.340.120.11−0.170.29),

and the solution is

(21) XS=(0.215981−0.09604060.101305−0.09604060.331082−0.1544870.101305−0.1544870.241782).

**Figure 1:**
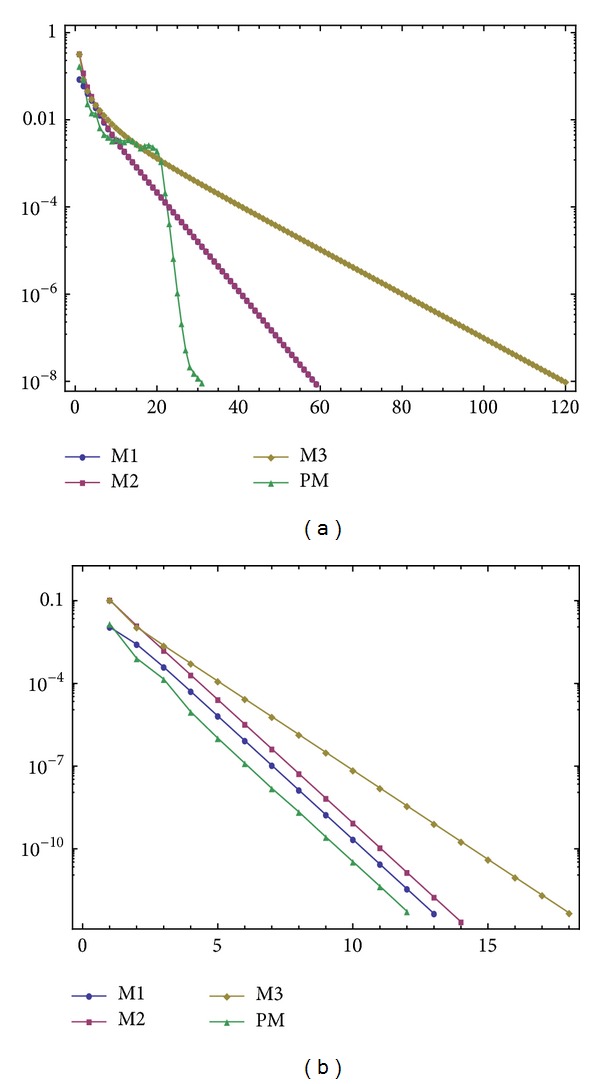
Number of iterations against accuracies for experiment 1 (a) and experiment 2 (b).

The results are given in Figure 1(a) in terms of the number of iterations when the stopping criterion is ||*X*_*k*+1_−*X*_*k*_||_*∞*_ ≤ 10^−8^.

Note that PM and M2 converge to *X*_*S*_, whereas all the other schemes converge to *X*_*L*_; thus we use other schemes to find the maximal solution of the dual equation *D*(*X*) = *X* + *AX* ^−1^ A* − *I* in our written codes so as to have fair comparisons. Here *t* = 2 has been chosen for PM (with *l* = 19). This *l* = 19 for the number of iterations in the inner finite loop of Algorithm 1 has been considered in the numerical report.

Furthermore, we have chosen this number empirically. In fact, varying *l* shows us that we even can obtain better or worse results than the reported ones in different examples.

In Example 1, we have used *t* = 2. In fact we have chosen this value for *t* since we are solving an operator equation in essence. To be more precise, we wish to consider the solution of the operator equation to be of multiplicity 2. This consideration makes the algorithm converge faster at the initial phase of the process and when we are enough close to the solution, then we flash back to the ordinary methods, that is, treat the solution as a simple zero (solution) of the operator equation.

**Example 2 (see [19]).**

Applying the stopping criterion ||*X*_*k*+1_−*X*_*k*_||_*∞*_ ≤ 10^−12^, we compare the behavior of various methods for the following test matrix:

(22) A=(0.1−0.15−0.25980760.150.2125−0.06495190.2598076−0.06495190.137),

with the solution as

(23) XS=(0.112684−0.00001301610.000142799−0.00001301610.0784090.01983070.0001427990.01983070.101129).

The results are illustrated in Figure 1(b), wherein *t* = 1.2 has been chosen for PM (with *l* = 1).

## 4. Conclusions

We have studied the fact that the minimal HPD solution of (1) is equivalent to the roots of a nonlinear map. This special map has been solved by the well-known Chebyshev method as a matrix inversion problem.

The developed method requires the computation of one matrix inverse at the beginning of the process and it is hence an inversion-free method. The convergence and the rate of convergence have been studied for this scheme. Furthermore, using a proper acceleration technique from the literature, we have further speeded up the process of finding the HPD solution of (1).

## Figures and Tables

**Algorithm 1:**
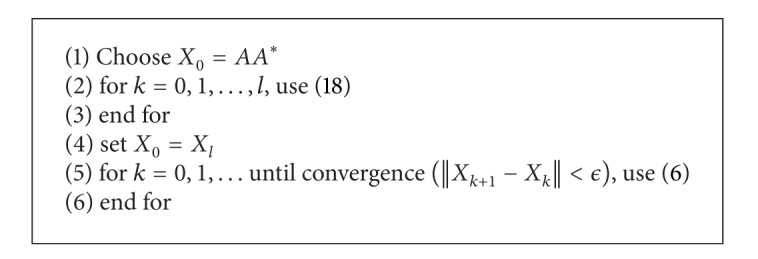
A hybrid method for computing the minimal HPD solution of (1).
